# Invasive treatment of pain associated with pancreatic cancer on different levels of WHO analgesic ladder

**DOI:** 10.1186/s12893-016-0136-3

**Published:** 2016-04-18

**Authors:** Łukasz Dobosz, Tomasz Stefaniak, Małgorzata Dobrzycka, Jagoda Wieczorek, Paula Franczak, Dominika Ptaszyńska, Katarzyna Zasada, Peter Kanyion

**Affiliations:** Department of General, Endocrine and Transplant Surgery, Medical University of Gdansk, ul. Smoluchowskiego 17, 80-214 Gdansk, Poland

**Keywords:** Splanchnicectomy, Pancreatic cancer, Pain

## Abstract

**Background:**

Pancreatic cancer is a malignant neoplasm with a high mortality rate, often associated with a delayed diagnosis, the early occurrence of metastasis and an overall, poor response to chemotherapy and radiotherapy. Pain management in pancreatic cancer consists mainly of pharmacological treatment according to the WHO analgesic ladder. Surgical treatment for pain relief, such as splanchnicectomy, is considered amongst the final step of pain management. It has been proven that splanchnicectomy is a safe procedure with a small percentage of complications, nevertheless, it is often used as a last resort, which can significantly decrease its effectiveness. Performance of thoracoscopic splanchnicectomy along the first step of the analgesic ladder may lead to long-lasting protection against the presence and severity of pain.

**Methods/Design:**

A prospective, open label, 1:1 randomized, controlled trial, conducted at a single institution to determine the effectiveness of invasive treatment of pain via splanchnicectomy, in patients with advanced pancreatic cancer. The size of tested group will consist of 26 participants in each arm of the trial, to evaluate the level of pain relief and its impact on quality of life. To evaluate the influence on patients’ rate of overall survival, a sample size of 105 patients is necessary, in each trial arm. Assessments will not only include the usage of analgesic pharmacotherapy throughout the course of disease, and overall patient survival, but also subjective pain perception at rest, in movement, and after meals (measured by NRS score questionnaire), the patient’s quality of life (measured using the QLQ-C30 and FACIT questionnaires), and any pain-related suffering (measured with the PRISM projection test). The primary endpoint will consist of pain intensity. Questionnaires will be obtained upon the initial visit, the day of surgery, the day after surgery, as well as during long-term follow-up visits, held every two weeks thereafter.

**Discussion:**

Earlier implementation of invasive treatment, such as thoracoscopic splanchnicectomy, can provide a higher efficacy of pain management, prevent deterioration in the patient’s quality of life, and lengthen their overall survival.

**Trial registration:**

ClinicalTrials.gov identifier: NCT02424279. Date of registration January 2, 2015.

## Background

Difficulties in the treatment of pancreatic cancer are frequently connected to the fact that the diagnosis is often made in the late stages of the disease. Total resection of the lesion is the treatment of choice, but is possible only in less than 20 % of cases [1]. The course of the disease in pancreatic cancer is extremely dramatic, with only 16 % of patients still alive 1 year after diagnosis. The average 5-year survival rate in patients with pancreatic cancer drastically decreases to a mere 5 % [2, 3]. Patients often suffer from intense chronic pain which leaves them severely debilitated, leading to a significant deterioration in their quality of life [4].

**Table 1:** Plan of proceedings

Group	Initial visit: NRS, QLQ-C30, FACIT, PRISM	Surgery	The measurement of pain intensity: 0 day after surgery	The measurement of pain intensity: 1 day after surgery	Follow up (every 2 weeks): NRS, QLQ-C30, FACIT, PRISM	Patient survival
Splanchnicectomy	+	+	+	+	+	+
NIPC	+				+	+

One of the available methods for the treatment of pain associated with pancreatic cancer, is thoracoscopic splanchnicectomy. It has been proven that splanchnicectomy is a safe procedure, with only a small amount of complications [5, 6], nevertheless, it is often used as a last resort in pain management, which can significantly decrease its effectiveness [7]. In a review of the current literature, there are many trials evaluating the safety and efficacy of splanchnicectomy in both patients with chronic pancreatitis and late-stage pancreatic cancer; however, there are no similar randomized trials about the effectiveness of this treatment in the early stages of pancreatic cancer, for patients with little to no complaints of pain.

### Hypothesis

The use of invasive treatment, such as thoracoscopic splanchnicectomy, as part of the first step of the analgesic ladder, can lead to long-lasting protection against the presence and severity of pain, help maintain a satisfactory quality of life despite disease progression, or may even extend the patient’s total survival time due to a reduction in the use and thus decrease in the adverse side effects of analgesic pharmacotherapy, such as the immunosuppressive effect of opioids and common post-prandial ailments leading to decreased nutrient intake.

## Methods and design

### Trial design

This trial is a prospective, open label, 1:1 randomized, controlled trial, conducted at a single institution. The aim of this study is to determine the effectiveness of invasive treatment of pain via splanchnicectomy, in patients with advanced pancreatic cancer. Assessments will not only include the usage of analgesic pharmacotherapy throughout the course of disease, and overall patient survival, but also subjective pain perception at rest, in movement, and after meals (measured by NRS score questionnaire), the patient’s quality of life (measured using the QLQ-C30 and FACIT questionnaires) [8, 9], and any pain-related suffering (measured with the PRISM projection test) [10, 11]. In addition, we intend to ascertain if earlier qualification for splanchnicectomy (upon lower steps of the WHO analgesic ladder) allows for a better therapeutic effect with this type of pain management.

**Fig. 1:**
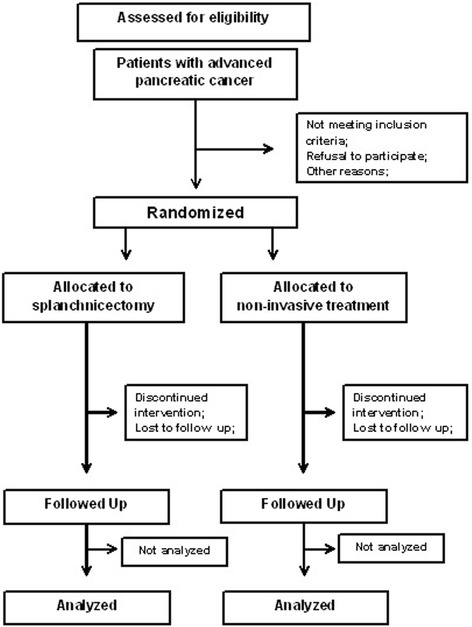
Flow diagram

A flow diagram of the trial is depicted in Fig. 1.

### Ethics and permissions

The final protocol was approved by the independent ethics committee of the Medical University of Gdansk (approval number NKBBN/216/2014). Informed consent will be obtained from the patient in both oral and written form prior to inclusion in the clinical trial.

### Patient evaluation and selection

Patients of both sexes with unresectable pancreatic cancer (determined upon intraoperative findings of local lesions or distant metastases, radiological advancement, or recurrence of local lesion and/or distant metastases) will be considered for inclusion in the study. All tumors of the pancreas will be confirmed histologically. Patients after prior surgical treatment of pancreatic cancer with radical intent will also be allowed to join the study. Patients will be enrolled in the study despite their incidence of pain symptoms and/or the intensity of pain therapy used, according to the WHO analgesic ladder steps 1, 2, or 3.

In Poland, according to the National Cancer Registry, about 3,000 patients are diagnosed with pancreatic cancer each year. In our Clinic, we treat about 50 patients per year with this type of carcinoma. We assume that a time period of 24 months will be sufficient enough to enroll a statistically significant group of patients in our trial.

### Inclusion criteria

- Patients with diagnosed pancreatic cancer: both non-operative tumor (intraoperative statement of organ and/or vessel infiltration, with local or distant metastases, radiological statement of progression, or local recurrence of tumor and/or distant metastases) and/or after prior surgical treatment.
- Age over 18 years.
- Signed informed consent to participate in the study.

### Exclusion criteria

- Age under 18 years.
- Intellectual inadequacy to complete necessary questionnaires.
- Coexistence of a disease or disease state in which there is significant chronic pain, which has been identified before the onset of pancreatic cancer.

### Setting

The setting of the study is the Department of General, Endocrine and Transplantation Surgery within the Medical University of Gdansk, Poland.

### Study design

#### Registration and randomization procedure

Prior to participation in the trial, patients determined as potential candidates will be informed about the purpose of the study and its implementation. In addition, each person will receive detailed information about the trial in writing. Patients will be allowed to review the methodology of the project and discuss any possible questions with their doctor, before they decide to take part in the trial. During their initial visit at the Clinic, participants will again go over the principles of the study. Upon agreement of both the doctor and patient for inclusion in the clinical trial, the patient must then state their intent to enroll in the study, both verbally and with a written consent form. If the patient is currently taking any analgesic pharmacotherapy, a grade according to the WHO analgesic ladder will be assigned to them [12]. Patients will then be randomly assigned to one of two groups. 1:1 Randomization will be generated for all strata by computer (Urbaniak, G. C., & Plous, S. (2013). Research Randomizer (Version 4.0) [Computer software]. Retrieved on June 22, 2013, from http://www.randomizer.org/). The first group will be offered the invasive treatment of pain with splanchnicectomy through a thoracoscopic approach, while the second group will be offered non-invasive conservative treatment, with the most appropriate available non-invasive pain control (NIPC). At each stage of the clinical trial, patients will be able to convert from the non-invasive intervention group to that of the group with invasive treatment (splanchnicectomy). However, these patients will then be excluded from participation in the clinical trial.

### Interventions

#### Surgical treatment

Each patient in the invasive intervention group will undergo a thoracoscopic splanchnicectomy. Prior to surgery, all electrolyte disturbances and fluid balance are corrected. A 1000 mg dose of Cefazolin is given as a single dose, thirty minutes prior to surgical incision as antibiotic prophylaxis. The surgery is performed under general anesthesia with endotracheal ventilation. The patient is placed in the flank thoracotomy position, with the initial procedure done on the patient’s left side. The skin and subcutaneous tissue is anesthetized locally with the injection of 0.5 % Bupivacine and then, by the use of two 5 mm ports, access to the left pleural cavity is obtained after lung collapse. Block of T6, T7, and T8 intercostal nerves is performed. The pleural cavity is insufflated with carbon dioxide and maintained at 8 mmHg throughout the procedure. The greater splanchnic nerve is identified at its origin in the sympathetic trunk, then isolated along with its lateral branches to the level of diaphragm and resected. Additional splanchnic nerves may be incised or excised if they are found connected to the greater splanchnic nerve. A pleural drain is inserted for a short period of time during desufflation, and is then removed. The incision points of the ports are closed with single sutures placed on the skin. The same procedure is performed on the patient’s right side. There is no histopathological examination of the excised tissue. Patients are given routine postoperative pain management. The procedure is recorded for future assessment. Patients are allowed to leave the hospital and return home on the first postoperative day.

Bilateral thoracoscopic splanchnicectomy has been shown to be a safe and effective procedure with a minimal mortality rate. It ensures precise visualisation of the splachnic nerves endoscopically without the need for a thoracotomy, which minimises blood loss during the procedure. Among the complications that can occur after this procedure, intercostal neuralgia is the most common, and occurs in about 25 % of cases. Less than 2 % of patients can suffer from other complications such as pulmonary atelectasis, chylothorax and orthostatic hypotension.

### Conservative treatment

Patients in the non-invasive, conservative treatment group are treated with the most appropriate available non-invasive pain control (NIPC). The therapy is conducted according to WHO and IASP (International Association for Study of Pain) guidelines. Pharmacotherapy is administered in accordance with the WHO analgesic ladder. The first step medications include non-opioid analgesics: paracetamol, ibuprofen, diclofenac, indometacine, and naproxen. In the second step, drugs from first step are still used, along with mild opioids such as codeine, and tramadol. Finally, the third step includes drugs from second step as well as strong opioids: morphine, fentanyl, oxycodon, and pethidine. Oral or percutaneous administration is the preferred route of administration as opposed to intravenous, subcutaneous or intramuscular administration. The analgesics are given in time-contingent basis. The patient is transferred to next step of the pain management ladder if their pain is stronger than a 6 in the NRS scale and/or if it continues for more than 5 days.

### Follow-up

Measurements of pain score (collected using the NRS - Numeric Rating Scale and the BPI - Brief Pain Inventory [13]) will be obtained upon the patient’s initial visit to the clinic and for the first group of patients (invasive treatment), prior to surgery, during the early postoperative period and on the first day after surgery, both in the primary location of pain (upper abdomen, epigastrium) and in the incision sites of the trocars.

After the initiation of treatment for both the invasive and non-invasive groups, the severity of pain will be measured in each group at two-week intervals during the patient’s follow-up visits in the clinic, or at the patient’s home, if there constitutes a physical or organization impediment to the patient’s abilities to participate in follow-up care. These visits will continue indefinitely, or until the patient’s death. All the measurement points are shown in Table 1.

### End points

#### Primary end points

- Pain intensity (NRS score questionnaire).

### Secondary end points

- Pain impact on quality of life (QLQ-C30 and FACIT questionnaire).
- Perception of the illness, suffering (PRISM questionnaire).
- Total need of analgesics.
- Total lifespan, overall survival time.
- The effectiveness of splanchnicectomy performed on early stages of pancreatic cancer.
- Postoperative complications.

### Sample size calculation (power of the study)

Analyzing the power of the study, we estimate that a test group of 26 participants in each arm of the trial will be required to be able to evaluate the level of pain relief and its impact on quality of life. To evaluate the influence on the patients’ rate of overall survive, a sample size of 105 patients is necessary, in each trial arm.

### Statistical analysis

Continuous data will be presented as the mean ± standard deviation and the range. Continuous variables will be compared between the two groups using an unpaired-sample student *t* test and Mann–Whitney test. Parametric variables will be analyzed using ANOVA and non-parametric variables will be analyzed with chi-square test. Statistical analysis will be performed using Statistica 11 PL licensed to the Medical University of Gdansk, in Poland. Results will be considered statistically significant for p < 0.05. An interim analysis will be performed after 52 patients have been randomized and treated.

## Discussion

Pancreatic cancer is a malignant neoplasm with a high mortality rate, often associated with a delayed diagnosis, the early occurrence of metastasis and an overall, poor response to chemotherapy and radiotherapy [2, 14]. A majority of patients are diagnosed with pancreatic cancer already in the advanced stages, where tumor resection is not a possible method of treatment. The average survival in unresectable tumors is currently 5.8 months and for those patients with resectable lesions, the average survival extends to about 12–15.9 months from the time of initial diagnosis [14].

Pancreatic cancer is a disease associated with severe chronic pain that leads to a dramatic worsening of the patient’s quality of life [15]. Upon initial diagnosis, already 75 % of patients admit to experiencing pain, and during the further progression of the disease, that percentage grows to over 90 % of patients with pancreatic cancer [16]. It has been established that a patient’s perception of pain intensity correlates with their overall survival, thus proving that pain management can be a remarkable challenge and demands an interdisciplinary approach [17]. The origin of pain caused by pancreatic cancer can be somatic, visceral or neuropathic. It can be induced by damage to healthy tissues mediated by the cancer cells, or due to the organism’s inflammatory response to the disease, even obstruction of the pancreatic duct, or by infiltration of the neoplasm into the surrounding tissues, especially that of the nerves and ganglia. Splanchnic pain impulses are transmitted by the sympathetic nerves to the splanchnic plexus and the sympathetic ganglions (Th12-L2). These nerve impulses are conveyed to the posterior cornua (T5-T12), and then continue their conduction to specific pain perception areas in the central nervous system [18].

The main basis for the treatment of chronic pain in pancreatic cancer is the WHO analgesic ladder, which is divided into three stages of intensity, depending on the pharmacotherapy used by the patient [12]. The medications used in the first step include non-opioid analgesics: paracetamol, ibuprofen, diclofenac, indometacine, and naproxen. In the second step, drugs from first step are still used, along with mild opioids such as codeine, and tramadol. Finally, the third step includes drugs from second step as well as strong opioids: morphine, fentanyl, oxycodon, and pethidine. However, it has been established that conservative treatment with analgesics, in many cases, does not allow for adequate analgesia. In addition, is often associated not only with tolerance, but also with the emergence of numerous adverse events such as pruritus, constipation, drowsiness, and impaired social interactions [18, 19]. Experimental data has demonstrated the superiority of pain prevention (pre-emptive analgesia) rather than that of reactive treatment of pain, due to the fact that prolonged activation of nociceptive pathways can lead to an activity-dependent plasticity, resulting in an increased response to the stimuli of pain and thus, the ineffectiveness of treatment [20, 21].

The fourth stage of the analgesic ladder proposes surgical treatment for the management of pain. It is possible to interrupt the conduction of pain impulses along the pain tract at the level of the celiac plexus (celiac plexus block) or splanchnic nerves (splanchnicectomy) [16].

However, present surgical methods such as celiac plexus block or thoracoscopic splanchnicectomy are considered to be reserved only for cases refractory to pharmacological analgesic treatment up to the third level of the analgesic ladder [22]. It has been demonstrated in the literature, that thoracoscopic splanchnicectomy is associated with a similar morbidity and mortality rate as performing a celiac plexus block. Splanchnicectomy has also been correlated with a longer-lasting analgesic effect, leading to a successful reduction in pain sensation and thus improvement of the patient’s quality of life, both in patients with chronic pancreatitis and those with pancreatic cancer [23–26]. Moreover, it appears that undergoing splanchnicectomy before the onset of pain, can in turn, offer a higher efficacy of future pain management therapies and even prolong a patient’s overall survival [7]. Therefore, it should be strongly emphasized that earlier implementation of thoracoscopic splanchnicectomy requires further thorough investigation as a modality for initial pain management.

## Abbreviations

NIPC: most appropriate available, Non-Invasive Pain Control
NRS: Numeric Rating Scale
